# Supplementary material for: CD47 Blockade Reprograms the Monocyte-Macrophage Axis to Promote Inflammation Resolution in Atherosclerosis
Source: bioRxiv. 2026 Apr 27:2026.04.24.720546. Preprint. [Version 1] doi: 10.64898/2026.04.24.720546 (PMC13142396; doi:10.64898/2026.04.24.720546)
Supplement: Supplement 1 [file NIHPP2026.04.24.720546v1-supplement-1.pdf]

bioRxiv preprint doi: https://doi.org/10.64898/2026.04.24.720546; this version posted April 27, 2026. The copyright holder for this preprint (which was not certified by peer review) is the author/funder, who has granted bioRxiv a license to display the preprint in perpetuity. It is made available under aCC-BY 4.0 International license.

1 Table S1. FACS Antibodies.

**Antigen**	**Fluorophore**	**Clone**	**Provider**	Catalog Nr.
CD45	PerCP Cy5.5	30-F11	BD Biosciences	561869
TER-119	BV510	TER-119	Biolegend	116237
CD45	BV510	30-F11	Biolegend	103138
CD11b	Pacific Blue	M1/70	Biolegend	101224
Ly6C	PE	HK1.4	Biolegend	128008
Ly6G	PE-Cy5	1-A8	Biolegend	127672
F4/80	PE-Cy7	BM8	Biolegend	123114
CD11c	BV605	N418	Biolegend	117333
CX3CR1	BV711	SA011F11	Biolegend	149031
CD45	APC-Cy7	30-F11	Biolegend	103116
CD11c	BV510	N418	Biolegend	117353
Ly6G	BV605	1-A8	Biolegend	127639
CD115	PE-Cy7	AFS98	Biolegend	135523

2

bioRxiv preprint doi: https://doi.org/10.64898/2026.04.24.720546; this version posted April 27, 2026. The copyright holder for this preprint (which was not certified by peer review) is the author/funder, who has granted bioRxiv a license to display the preprint in perpetuity. It is made available under aCC-BY 4.0 International license.

Table S2. Primer sequences.

**Gene**	**Forward**	**Reverse**
Gapdh	GTGTTCCTACCCCCAATGTGT	ATTGTCATACCAGGAAATGAGCTT
Il1b	TGACAGTGATGAGAATGACCTGTTC	TTGGAAGCAGCCCTTCATCT
Il5	ATGGAGATTCCCATGAGCAC	AGCCCCTGAAAGATTTCTCC
Il6	GCTACCAAACTGGATATAATCAGGA	CCAGGTAGCTATGGTACTCCAGAA
Il13	CCTCTGACCCTTAAGGAGCTTAT	CGTTGCACAGGGGAGTCT
Il23	TCCCTACTAGGACTCAGCCAAC	AGAACTCAGGCTGGGCATC
Tnf	CGGAGTCCGGGCAGG	GCTGGGTAGAGAATGGATGAA
Ccl2	CAGCCAGATGCAGTTAACGC	GCCTACTCATTGGGATCATCTTG
Ccl5	CAGCAGCAAGTGCTCCAATC	CACACACTTGGCGGTTCCTT
Tgfb	ATACCAACTATTGCTTCAGCTCCACAG	GTACTGTGTGTCCAGGCTCCAAATAT
Cx3cl1	ACGAAATGCGAAATCATGTGC	CTGTGTCGTCTCCAGGACAA
Vcam1	GCCACCCTCACCTTAATTGCTATG	TGTGCAGCCACCTGAGATCC
Icam1	AACTGTGGCACCGTGCAGTC	AGGGTGAGGTCCTTGCCTACTTG

bioRxiv preprint doi: https://doi.org/10.64898/2026.04.24.720546; this version posted April 27, 2026. The copyright holder for this preprint (which was not certified by peer review) is the author/funder, who has granted bioRxiv a license to display the preprint in perpetuity. It is made available under aCC-BY 4.0 International license.

Supplemental Figures

Figure S1. Gating strategy for CD45$^+$ aortic leukocytes and systemic characterization of the prevention model.

A, Representative flow cytometry gating strategy to isolate viable aortic CD45$^+$ cells, excluding BV510-labelled circulating leukocytes (SYTOX$^-$ BV510$^-$ PerCP-Cy5.5$^+$). **B,** Body weight monitoring during the experimental period of the prevention model (n=12 IgG; n=13 anti-CD47). **C** and **D,** Heart (**C**) and spleen (**D**) weight at euthanasia (n=24 IgG; n=24 anti-CD47). **E,** Quantification of total cholesterol (n=22 IgG; n=21 anti-CD47), high-density lipoprotein (HDL) (n=21 IgG; n=19 anti-CD47), low-density lipoprotein (LDL) (n=21 IgG; n=21 anti-CD47), and non-fasting glucose (n=22 IgG; n=21 anti-CD47) in blood. **F,** t-SNE plot of publicly available scRNA-seq data from aortic CD45$^+$ leukocytes of mice fed chow diet (CD) and Western Diet (WD) (Cochain et al., 2018). **G**, t-SNE plot of the fully integrated, batch-corrected dataset showing distinct cell clusters.

Data and error bars present mean ± SD for parametric and median ± IQR for non-parametric results. Statistical analysis was performed using two-way ANOVA, Mann-Whitney U test (two-tailed) and unpaired Student's *t*-test (two-tailed). All data and statistical analysis are provided in Source Data.

bioRxiv preprint doi: https://doi.org/10.64898/2026.04.24.720546; this version posted April 27, 2026. The copyright holder for this preprint (which was not certified by peer review) is the author/funder, who has granted bioRxiv a license to display the preprint in perpetuity. It is made available under aCC-BY 4.0 International license.

Figure S2. Monocyte subclusters.

A, Monocle3 pseudotime trajectory of monocyte-macrophage populations superimposed on a t-SNE plot (left) and heat map representing the differentiation progression of the top 20 genes along pseudotime and monocyte-macrophage subclusters (right). **B,** Expression of 5 selected marker genes per monocyte subclusters. The color scale represents log-transformed gene expression. **C**, Dot plot depicting 3 selected marker genes used for monocyte subcluster identification.

bioRxiv preprint doi: https://doi.org/10.64898/2026.04.24.720546; this version posted April 27, 2026. The copyright holder for this preprint (which was not certified by peer review) is the author/funder, who has granted bioRxiv a license to display the preprint in perpetuity. It is made available under aCC-BY 4.0 International license.

Figure S3. Aortic myeloid cell gating strategy and validation of inflammatory gene expression in the prevention model.

A, Representative flow cytometry gating strategy to analyze major myeloid cells in the aorta. Live, single, CD45$^+$ cells were pre-gated to identify CD11b$^+$ myeloid cells, Ly6G$^+$ neutrophils, Ly6C$^+$ monocytes, CD11c$^+$ dendritic cells, and F4/80$^+$ macrophages. **B**, Flow cytometric analysis of Ly6C^{hi} and Ly6C^{lo} monocytes in aorta-draining lymph nodes (n=10 IgG; n=9 anti-CD47) and bone marrow (n=18 IgG; n=19 anti-CD47). **C,** scRNA-seq gene expression of *Nfkb1 and Nfkbiz* in the MPC cluster. **D,** qPCR analysis of cytokines/chemokine expression (*Tnf, Il5, Il13, Il23, Cx3cl1)* and adhesion molecules (*Vcam1, Icam1)* in aortic arch samples (n=7 IgG; n=5-7 anti-CD47).

Data and error bars present mean ± SD for parametric and median ± IQR for non-parametric results. Statistical analysis was performed using unpaired Student's *t*-test (two-tailed), unpaired Welch's *t*-test (two-tailed) and Mann-Whitney U test (two-tailed). All data and statistical analysis are provided in Source Data.

bioRxiv preprint doi: https://doi.org/10.64898/2026.04.24.720546; this version posted April 27, 2026. The copyright holder for this preprint (which was not certified by peer review) is the author/funder, who has granted bioRxiv a license to display the preprint in perpetuity. It is made available under aCC-BY 4.0 International license.

Figure S4. Intervention model cluster representation.

A, Additional Oil Red O (ORO) staining images depicting lesion size analysis (related to Figure 3B). Scale bar, 250 µm. **B**, Expression of 6 selected marker genes per major cell cluster in the intervention scRNA-seq (left) and dot plot depicting 5 selected marker genes used for identification (right). The color scale represents log-transformed gene expression. **C,** Expression of 8 selected marker genes per MPC + APC subclusters in the intervention model (left) and dot plot depicting 5 selected marker genes used for MPC + APC subcluster identification (right). The color scale represents log-transformed gene expression.

bioRxiv preprint doi: https://doi.org/10.64898/2026.04.24.720546; this version posted April 27, 2026. The copyright holder for this preprint (which was not certified by peer review) is the author/funder, who has granted bioRxiv a license to display the preprint in perpetuity. It is made available under aCC-BY 4.0 International license.

Figure S5. CD47 blockade reactivates efferocytosis machinery in leukocytes.

A, Volcano plot showing DEGs in $CD45^+$ leukocytes following treatment with anti-CD47 versus IgG control. Significant hits (FDR $<$ 0.10; log2FC $>$ 0.5) are color-coded: blue indicates downregulation and red indicates upregulation. Canonical efferocytosis-related genes are highlighted with black circles. **B** and **C,** Pathway analysis depicting significantly upregulated KEGG pathways (**B**) and biological processes (**C**) in anti-CD47-treated $CD45^+$ leukocytes. **D,** Gene set enrichment analysis (GSEA) of phagocytosis (GO:0006909) in myeloid cells after anti-CD47 treatment. E, Quantification of total cleaved caspase-3 area in aortic root sections, in relation to total vessel area (TVA, left) and lesion area (right).

bioRxiv preprint doi: https://doi.org/10.64898/2026.04.24.720546; this version posted April 27, 2026. The copyright holder for this preprint (which was not certified by peer review) is the author/funder, who has granted bioRxiv a license to display the preprint in perpetuity. It is made available under aCC-BY 4.0 International license.

Figure S6. CD47 blockade alters aortic and systemic monocyte dynamics.

A, Flow cytometric analysis of YG bead-labeled (top) and EdU-labeled (bottom) $Ly6C^+$ blood monocytes 24 hours after injection (n=6 IgG; n=6 anti-CD47). Population frequencies of EdU^+ and YG $Bead^+$ gates are depicted. **B,** Flow cytometric analysis of $CD45^+$ leukocytes, $CD11b^+$ myeloid cells, $Ly6G^+$ neutrophils, $F4/80^+$ macrophages and $CD11c^+$ dendritic cells in aorta (top) and blood (bottom) in the intervention model (n=8 IgG; n=9 anti-CD47). **C** and **D,** Additional representative immunofluorescence images of EdU-labeled $Ly6C^{hi}$ (**C**) and YG fluorescent bead-labeled $Ly6C^{lo}$ (**D**) monocytes. The white dashed line depicts neointima. Scale bars, 100 µm; inset, 10 µm (**C**) and 50 µm (**D**). **E**, Flow cytometric analysis of $CD115^+$ (M-CSF receptor, CSF1R) $Ly6C^+$ monocytes (n=6 IgG; n=6 anti-CD47). Population frequencies of $CD115^+$ cells are depicted. **F,** Additional representative immunofluorescence images of $CD68^+$ macrophages in aortic root sections. Scale bar, 250 µm. **G**, Additional representative immunofluorescence images of proliferating $Ki67^+CD68^+$ macrophages in aortic root sections. The white dashed line depicts neointima. Scale bar, 50 µm; inset 10 µm. **H**, Quantification of CX3CR1 expression by flow cytometry in splenic (top) and bone marrow (bottom) $Ly6C^{hi}$ and $Ly6C^{lo}$ monocytes (n=9 IgG; n=9 anti-CD47). Data are represented as mean fluorescence intensity (MFI).

Data and error bars present mean ± SD for parametric and median ± IQR for non-parametric results. Statistical analysis was performed using unpaired Student's *t*-test (two-tailed) and Mann-Whitney U test (two-tailed). All data and statistical analysis are provided in Source Data.

bioRxiv preprint doi: https://doi.org/10.64898/2026.04.24.720546; this version posted April 27, 2026. The copyright holder for this preprint (which was not certified by peer review) is the author/funder, who has granted bioRxiv a license to display the preprint in perpetuity. It is made available under aCC-BY 4.0 International license.

Figure S7. Ligand-receptor interaction analysis.

A, CellChat analysis of innate immune cell communications. The heat map depicts the max interaction strength per cell communication pair, acting as either sender (ligand) or receiver (receptor). **B,** Heat map showing the interaction strengths between macrophage clusters involving *Ccl2-Ccr2* signaling. **C,** CellChat analysis of the overall *Ccr-Ccl* interaction network between MPC-APC subclusters. **D,** Dot plot depicting ligand-receptor interaction strengths of the specific *Ccr-Ccl* pathway. **E,** Heat maps showing the number of interactions (left) and interaction strengths (right) between macrophage clusters involving *Gas6-Axl/Mertk* signaling.

bioRxiv preprint doi: https://doi.org/10.64898/2026.04.24.720546; this version posted April 27, 2026. The copyright holder for this preprint (which was not certified by peer review) is the author/funder, who has granted bioRxiv a license to display the preprint in perpetuity. It is made available under aCC-BY 4.0 International license.

Figure S8. Efferocytosis-competent macrophage states are conserved in human atherosclerotic plaques.

A, t-SNE plot of human coronary artery cells; the myeloid cell population is highlighted in the box. **B,** t-SNE representation of human coronary myeloid cells after subsetting and reclustering. **C,** Violin plot of AUCell scores for an apoptotic cell burden gene signature across human coronary myeloid cell clusters. **D,** Analysis workflow showing the curation of DEGs in mouse MPC + APC clusters following CD47 blockade and subsequent human ortholog conversion. Converted genes were separated into functional clusters to identify myeloid responders in human atherosclerotic plaques. **E,** Violin plots depicting AUCell scores for anti-CD47-enhanced efferocytosis/phagocytosis (left) and survival/homeostasis (right) gene sets in human coronary myeloid cells. **F,** AUCell score analysis of negative control gene sets, including metabolic, housekeeping, RNA processing, protein folding, and cytoskeleton-related genes.
